# Dipsomania and Forensic Medicine

**Published:** 1886-09

**Authors:** Edward C. Mann

**Affiliations:** Member of the Medical Society of the County of New York


THE RELATION WHICH DIPSOMANIA BEARS TO FORENSIC MEDICINE. *By* EDWARD C. MANN, M. D., *Member of the Medical Society of the County of New York.*

The present law holds drunkenness to be no excuse for crime. The disease of dipsomania is not drunkenness. The state of intoxication is merely one of many symptoms of the disease. True dipsomaniacs are totally irresponsible for acts committed immediately before, during and after attacks, just as epileptics are, because of their intellectual condition before the paroxysm of inebriety—on account of the impulsive character of their actions and on account of the toxic delirium with which it is often followed. The great reason why the dipsomaniac is not responsible is, because he is not master of his desire to drink. Even in his lucid intervals, the dipsomaniac is not a normal man, and there is a modified responsibility attached to all his acts. The dipsomaniac is a sick man to be cured, not a criminal to be punished.

Dipsomania, the great diagnostic mark of which is an irresistible craving for alcohol in some form, coming on at regular intervals of either day, week, month or year, as the case may be, may be hereditary and evolved with the natural evolution of a person's organism, or it may be due to postnatal conditions.

Paroxysms of dipsomania are always preceded by depressive insomnia and excessive nervousness, like the other insanities.

What, now, is the real essence of this disease? It is deranged organic or special sensation or impulse which manifests itself, not as in ordinary insanity, by suicidal or homicidal impulse, or by the morbidly erratic feeling which misleads the judgment and conduct of the kleptomaniac, pyromaniac or nymphomaniac, but owing to the changing and misleading subjective impressions of the dipsomania he is impelled against his will and judgment, by a blind, irresistible impulse, to seek in excessive indulgence in alcohol for the relief of this subjective morbid condition of his nervous system. The craving is beyond the control of the patient. He cannot help it. His drinking is the product of disease. He is misled and perverted in the exercise of his psychic powers by abnormal condition of his brain and centric nervous system. I have seen inebriety or dipsomania in its hereditary forms exhibit, 1st, an abnormal aptitude, requiring an additional excitant factor to develop it into active morbid expression; 2d, the inborn defect so great as to require only the natural organic evolution of growth to reveal it; and, 3d, still greater, not requiring even this to unfold it, I have seen it displaying itself in states of organic retrogression and nerve-instability, so extreme as to be perceptible at the earliest period when the display of mind is perceptible at all, idiocy, imbecility and infantile insanity.

Morbid organic conditions, therefore, lie at the root of this disease. Take the case of a lady where normal character is pure and correct, where intellect is vigorous, who in habit is most abstemious, who, as a rule, never drank anything stronger than tea or coffee or water. This woman becomes depressed without adequate cause, suffers from insomnia, suffers from great nervousness. Then comes on a craving or impulse for alcohol, and perhaps a fondness for low society. She drinks till she can drink no more, until intoxicated, perhaps drinks for several days; is then horror struck and filled with remorse, and is perhaps looked upon with horror by her nearest and dearest friends. Now, why has she done this? Surely not because she wanted the liquor and merely chose to indulge in alcohol to excess. She has obeyed an impulse or craving which she could not resist, which very likely she fought against. She could not successfully fight against the subjective morbid change in her organism which caused the craving. That has to be removed, if at all, by a careful course of medical treatment. That woman is not responsible either for her paroxysm of dipsomania nor for any overt act which she may commit in it, and she is liable to commit suicide, or homicide, or to become a kleptomaniac or to be nymphomaniacal. I have treated a score of such cases, and it behooves the law to respect medicine when the latter declares authoritatively that dipsomania is a disease, the subjects of which are laboring under a species of mental alienation, and not responsible beings. The subjects of this disease demand the protection of medicine and law, and not fine and imprisonment, and from my personal experience of fifteen years I cannot make my plea for them too earnest.

The disordered impulse or craving for alcohol, and, in like manner, for chloral and opium and some other drugs, dominates the character and morbidly masters the reason and the conduct. My experience and observation show the frequent interchangeability of inebriety and dipsomania with grave diseases of the nervous system, in which the neuropathic diathesis prevails. The dipsomaniac tendency of the constitution in some families seems vicarious, with the acknowledged or recognized neuropathic. I have frequently seen one branch of a family to show insanity, epilepsy or organic brain disease, and another to reveal the neuropathic taint in dipsomania. The chief triumphs in the therapeutics of dipsomania belong to the plan of treatment which places the patient under medical supervision and restraint, maintains the physiological tone of the vaso-motor system, and which re-establishes the perfect stability of the higher cerebral centers, the psycho-motor and the psychical, up to the point of their highest resisting power. Tobacco should be dropped, to cut off nicotine empoisonment, the hours of rest and labor carefully regulated, night work discontinued, sleep encouraged, and time be given to rest for brain and mind. Rest is a condition of nerve repair and power, and want of timely rest causes much brain disease and neurasthenia. Depression, insomnia and nervousness must all be removed by proper treatment, and a trained resistance of the nervous system to the disease be brought about. The patient must be kept under treatment until the nervous system becomes vigorous and well poised and adequately recuperated. A strong brain and a well nourished nervous system is the state of physiological antagonism to the disease of dipsomania. Everything that tends to undermine nerve stamina and induce nerve disintegration must be stopped, and recuperative conditions and physical regeneration brought about by all means in our power. We must teach our patient how to build his brain up instead of exhausting it. Inebriety occurring from post-natal causes is a preventable brain and nerve disease. Inebriates should be forbidden marriage, always, both by public opinion and by law, for inebriety will breed its like. We need to stamp out the hereditary descent of organically defective persons. The world needs sound brains and vigorous nervous systems, and progressive men should work to this end, so that dipsomania and kindred neuropathic diseases may be stamped out and suppressed.

As long, however, as the disease exists, let the unhappy subjects occupy their proper medico-legal relation, which is one of irresponsibility deserving protection.

SUNNYSIDE HOME FOR NERVOUS INVALIDS,
Brooklyn, N. Y.

